# Branded Foods Databases as a Tool to Support Nutrition Research and Monitoring of the Food Supply: Insights From the Slovenian Composition and Labeling Information System

**DOI:** 10.3389/fnut.2021.798576

**Published:** 2022-01-04

**Authors:** Igor Pravst, Maša Hribar, Katja Žmitek, Bojan Blažica, Barbara Koroušić Seljak, Anita Kušar

**Affiliations:** ^1^Nutrition Institute, Nutrition and Public Health Research Group, Ljubljana, Slovenia; ^2^Biotechnical Faculty, University of Ljubljana, Ljubljana, Slovenia; ^3^VIST–Faculty of Applied Sciences, Ljubljana, Slovenia; ^4^Computer Systems Department, Jozef Stefan Institute, Ljubljana, Slovenia

**Keywords:** food composition database, labeling, pre-packed, nutrition declaration, market, Slovenia, CLAS

## Abstract

Branded foods databases are becoming very valuable not only in nutrition research but also for clinical practice, policymakers, businesses, and general population. In contrast to generic foods, branded foods are marked by rapid changes in the food supply because of reformulations, the introduction of new foods, and the removal of existing ones from the market. Also, different branded foods are available in different countries. This not only complicates the compilation of branded foods datasets but also causes such datasets to become out of date quickly. In this review, we present different approaches to the compilation of branded foods datasets, describe the history and progress of building and updating such datasets in Slovenia, and present data to support nutrition research and monitoring of the food supply. Manufacturers are key sources of information for the compilation of branded foods databases, most commonly through food labels. In Slovenia, the branded food dataset is compiled using standard food monitoring studies conducted at all major retailers. Cross-sectional studies are conducted every few years, in which the food labels of all available branded foods are photographed. Studies are conducted using the Composition and Labeling Information System (CLAS) infrastructure, composed of a smartphone application for data collection and online data extraction and management tool. We reviewed various uses of branded foods datasets. Datasets can be used to assess the nutritional composition of food in the food supply (i.e., salt, sugar content), the use of specific ingredients, for example, food additives, for nutrient profiling, and assessment of marketing techniques on food labels. Such datasets are also valuable for other studies, for example, assessing nutrient intakes in dietary surveys. Additional approaches are also being tested to keep datasets updated between food monitoring studies. A promising approach is the exploitation of crowdsourcing through the mobile application VešKajJeš, which was launched in Slovenia to support consumers in making healthier dietary choices.

## Introduction

People's diets are composed of a wide variety of foods and drinks (further referred to as “foods)”. In general, we can distinguish between *generic* and *branded foods*. For example, orange juice could be considered a generic food, while on the market, there are a wide variety of branded orange juices, which can have notable differences in taste and nutritional composition. Such products are typically pre-packaged and labeled, and such food labeling information can be a useful resource for the compilation of branded foods composition databases.

It should be mentioned that the composition of foods is sometimes regionally specific, with notable differences between countries. Such differences are more expressed for some nutrients/foods than others. For example, while amino-acid composition in most meats is relatively stable, the content of many other nutrients can vary in foods available worldwide because of different cultivars and agricultural practices, differences in soils and climatic conditions (1) or consumer preferences specific to certain region. Therefore, composition databases for generic food are maintained in different countries and regions. This is also the case for processed branded foods, where between-country differences can be even more notable (2).

### Importance of Food Composition Information

Data about the composition of foods consumed in a population's diet is very important and has a wide variety of uses (Table 1). In research, the data can be used in epidemiological dietary studies to investigate nutrient intake and identify populations at risk for deficiencies. In clinical intervention trials, the datasets can be used to account for dietary factors, where diets/foods are co-founding factors in treatments. In food supply studies, the data can be used to investigate changes over time in the composition of foods. This data is also very important in clinical practice (i.e., in dietary counseling and preparation of diets for patients with special dietary needs or medical conditions). For policymakers, the data are used to set targets for reformulation, assess food reformulation programs and make evidence-based policy decisions. Companies use this data to identify opportunities for improving the composition of foods and to provide information for technology (IT) services, which use food composition data to support dietary, lifestyle, and health objectives. These data are also important for consumers to support informed selections of healthier foods and assure food safety, particularly those with special dietary needs, including allergies.

**Table 1:** Examples of the use of food composition and labeling information.

** 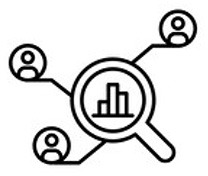 **	** 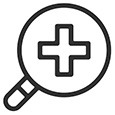 **	** 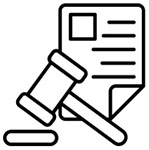 **	** 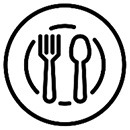 **	** 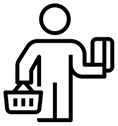 **
**Research**	**Clinical practice**	**Policymakers**	**Businesses**	**Consumers**
•Epidemiological dietary studies •Dietary intervention studies •Clinical intervention trials, where diet or foods are considered co-founding factors •Food supply studies •Assessment of exposure to food components	•Nutritional counseling in patients •Preparation of diets for patients with special dietary needs (including allergies) or medical conditions (for example, diabetes) •Identification of dietary risks	•Basis for evidence-based food policy decisions •Setting the targets for reformulation •Assessment of the efficacy of food reformulation programs •Regulatory restrictions related to specific food components (trans fats, additives)	•Identification of opportunities for improving the composition of foods •Comparisons with other foods–use of comparative nutrition claims •Promotion of foods with improved nutritional composition •Providers of IT services, where food composition data is used to support dietary, lifestyle, and health objectives	•Supporting the informed selection of foods •Enabling comparison of different foods •Supporting choices of healthier foods •Assuring food safety, particularly to those with special dietary needs (including allergies)

From a public health perspective, monitoring of food composition and labeling of branded foods available in any country provides insights into the market availability and informs the development of reformulation strategies as well as the planning, implementation, and monitoring of different public health interventions (3–5).

### Food Labeling and Composition Data

In the European Union, labeling of branded foods is regulated by Regulation (EU) No 1169/2011 on the provision of food information to consumers (6). In practice, we can distinguish between mandatory, conditionally mandatory, and voluntary food labeling information (examples provided in Supplementary Table 1).

A typical example of mandatory food labeling information is the ingredient list, which includes all the food ingredients in descending order of weight, as recorded at the time of their use in the manufacture of the food (6). This information is typically provided in unstructured text format (list), which can also contain embedded complex ingredients, including food additives. In some cases, ingredients in the ingredient list need to be quantified (QUID - Quantitative Ingredient Declaration). For example, if an ingredient appears in the name of the food or is usually associated with that name by the consumer, or if an ingredient is emphasized on the labeling in words, pictures, or graphics.

With a few exceptions, nutrition declaration is also considered mandatory food labeling information (6). In the EU values are typically expressed per 100 g or ml. The mandatory parts of the nutrition declaration include (a) energy value and the amounts of (b) fat, (c) saturates, (d) carbohydrate, (e) sugars, (f) protein, and (g) salt. However, other parts of the nutrition declaration are non-mandatory or conditionally mandatory, including the amounts of (a) mono-unsaturates, (b) polyunsaturates, (c) polyols, (d) starch, (e) fiber, and (f) vitamins or minerals (Table 2). Food manufacturers can decide to include this information on food labels using the regulated format (6). However, if such nutrients/constituents are mentioned in nutrition or health claims on the label, their inclusion in the nutrient declaration is conditionally mandatory (7).

**Table 2:** Composition-related information on labels of processed brand foods in the EU (6).

**Food composition information**	**Note**	**Mandatory information**
Ingredient list	Ingredients of the food in descending order of weight	✓
**Nutrition declaration**		
Energy	(kJ/kcal per 100 mg or mL)	✓
Fat		✓
- Saturates		✓
- Mono/poly-unsaturates		*x* ^*^
Carbohydrate		✓
- Sugars		✓
- Polyols/starch		*x*
Fiber		*x* ^*^
Protein		✓
Salt		✓
Vitamins & minerals	The units specified in regulation (6)	*x* ^*^

*Nutrition declaration information for specific nutrients/constituents are provided in grams per 100 mg or ml.*

* *Conditionally mandatory (if the constituent is mentioned in nutrition/health claim) (7)*.

### Potential Sources for the Compilation of Branded Foods Databases

Unlike less processed generic foods, the composition of processed pre-packaged ‘branded foods’ can change considerably in relatively short time periods because food manufacturers adapt formulations to address consumers' expectations and regulatory requirements, foods are removed from the market, and new ones are launched (8).

Datasets with branded food composition and labeling information originate from a variety of sources (Table 3).

**Table 3:** Typical sources of branded food composition and labeling information.

**Food manufacturers**	**Food monitoring**	**Crowdsourcing**
Sharing food composition information to databases (voluntarily)	Laboratory analyses of available foods (not feasible on a large scale) Data collections from food labels in food stores Web-scraping: data collections from online sources (online shops, web pages of food producers)	Enabling consumers to collect and share data on the composition of foods, i.e., through smartphone or web applications

#### Data Provided by Food Manufacturers

Data can be received directly from food manufacturers, but their participation in providing this food composition information to database compilers is voluntary. Food companies rarely decide to share this information to open databases, and if they do, they often only share some of the required information (e.g., information for nutritional labeling of products). There are some good examples of such a collaborative approach for creating branded food datasets (9–13), but very few capture the dynamic changes of the food market (13). The progress in the standardization of this area also needs to be noted. Branded foods are usually labeled with EAN/UPC barcodes (14), which are designed for a high-volume scanning environment and are therefore suitable for a retail point-of-sale (POS) system. EAN-13 barcodes are mostly used in Europe. Each barcode is linked with a unique product identifier, usually the GS1 Global Trade Item Number (GTIN) (15). The food name is considered a mandatory attribute in the GTIN Register, while food composition data are not included. It should be mentioned that GS1 also maintains the Global Data Synchronization Network (GDSN), a globally operating, standardized network that enables the exchange of all types of master product data, including food labeling and composition data, between brand owners, manufacturers, suppliers, distributors, and retailers (16). In the GDSN, based on the Global Data Model (17), specific food labeling parameters are considered mandatory information; however, the use of GDSN is voluntary, meaning that not all items in the GTIN Register have a corresponding record in the GDSN. Furthermore, the GDSN record is only available for subscribers and is not open access. Also, some challenges related with EAN barcode numbers also need to be mentioned. While majority of branded foods have assigned standardized GTIN EAN barcodes, some retailers are using their own non-standardized barcoding systems. In such systems similar products (but with different nutritional composition) sometimes have same barcode number. For example, different flavors of same brand/packaging of fruit yogurt can have same barcode number. There are also some challenges if standardized GTIN EAN barcodes are used, because some products can receive new barcode number without any change in the composition, while in some cases manufacturers do not change barcode number even in case or major change in the composition.

### Food Monitoring Studies

Food monitoring studies typically refer to gathering information about foods available in the food supply in any given market (country and region) using a variety of methods. Considering that chemical analysis of thousands of foods is not a feasible option, a typical approach for collecting branded food information is cross-sectional food monitoring studies in food stores, where data is extracted from food labels (18–20). However, to estimate nutritional composition food labeling data is usually calculated based on the ingredients list, rather than gained by chemical analyses; consequently labeling data can be prone to variety of errors such as batch-to-batch variability, incorrect calculations, processing and stability issues (21). Mandatory food composition information on food labels (Table 2) is a particularly important resource for compiling food composition databases because such information can be subject to official controls by food authorities (inspection). It should also be noted that tolerances for the control of compliance of nutrient values declared on food labels have been established (22).

A methodological approach for conducting food monitoring studies was established within the GFMG (Global Food Monitoring Group) (19) and INFORMAS (International Network for Food and Obesity/Non-communicable Diseases (NCDs) Research, Monitoring and Action Support) initiatives in 2013 and 2015, respectively (23, 24). These protocols support regular (preferably yearly) regional data collection on food composition and labeling in all major food suppliers, preferably including photographs, across all available food categories. However, this approach is hardly feasible in practice because such data collection is expensive and time-consuming. Therefore, food monitoring studies are commonly conducted with partial data collection focusing on selected food categories and in selected food shops and are not performed very often (2).

While harmonized data collection enables relevant international comparisons of the food supply (2, 25), such cross-sectional food monitoring studies face several challenges, in particular, keeping the database up to date with newly launched and reformulated products (8, 13). Additional methods are therefore important to enable more frequent data collections.

Online food stores are a promising additional resource for the compilation of branded food databases. While most foods are still sold in classic food stores (i.e., supermarkets), online shops are gaining importance. Most recently, the COVID-19 epidemic increased the use of online food purchases (26), which resulted in the further development of online stores and more foods available by delivery. While e-shops sell fewer foods than classic supermarkets, it is expected that market-leading brands are available online, making such an environment interesting for data collection. Recently, an automated big data analysis approach was applied in the UK to exploit the potential of this market. Harrington et al. tested the extraction of food products' data from supermarkets' webpages weekly (27), allowing timely observations of changes in the marketplace.

#### Collection of Data Using Crowdsourcing

Although there are several definitions of crowdsourcing, in general, this term is used for outsourcing different tasks to a crowd of people to complete them; the crowdsourcer can be an organization or an individual (28). Crowdsourcing is an innovative approach used for tasks where a vast amount of data needs to be collected and, therefore, can benefit from a larger group of people completing the task. Advantages include reduced costs, speed, flexibility, scalability, diversity, and participation of citizens, while disadvantages are related to accuracy and duplication. While crowdsourcing has rapidly developed in informational sciences, its application in public health and nutrition is also very promising (29, 30). With the rise of the usage of the internet, an innovative way to access and interpret different types of information became widely available also to consumers. Such platforms can commonly serve as an interface to collect crowdsourcing data.

In the context of food monitoring, these emerging new methods and associated technologies create new opportunities to keep up with the rapid changes in the food chain with less effort. A method of data collection using crowdsourcing allows capturing food market changes more regularly and at a considerably reduced cost while simultaneously delivering value for end-users or crowdsources. Examples of web-based crowdsourcing platforms are Open Food Facts (31) and The Open Food Repo (32) initiatives, which enable the collection of food composition data from different countries. The advantage of such an approach is that created datasets can be used to investigate differences in food composition on global markets (33–35).

The increased use of smartphones and their technical capacities also affect the potential of crowdsourcing in this area considerably. The Australian FoodSwitch application for smartphones was developed to collect branded food composition information and has yielded impressive results (36). A particular innovation in the application was incorporating a crowdsourcing function whereby users are able to contribute information on missing or new products. The crowdsourcing tool within the mobile application has enabled a substantial expansion of the underlying database. This shows that an extensive volume of crowdsourced data provides effective real-time, inexpensive tracking of the nutritional composition of foods in the food supply (37) and reveals a unique opportunity for using such an approach in other countries (38).

#### Future of Branded Food Datasets

There is no doubt that with developments in this scientific field and progress in information technology, branded foods datasets will gain importance in the future. It should be mentioned that existing datasets and food nutrition security data in general, although widespread, are fragmented and lack critical mass and accessibility. The data are not readily found, accessible, interoperable, or reusable (FAIR). The European project Food Nutrition Security (FNS) Cloud (https://www.fns-cloud.eu/; accessed 11.10.2021), funded by the European commission HORIZON2020 framework program, is addressing this challenge. The FNS-Cloud is developing the first generation ‘food cloud’ by federating existing and emerging datasets and developing and integrating services to support re-use through the European Open Science Cloud (EOSC). The project's major objectives are to support standardization and demonstrate the usability of such datasets (39).

Food manufacturers are key data sources for the future of branded food composition databases and should be encouraged to share food composition data in open-access databases. Major progress could be achieved if this became a legal requirement. Despite the above-mentioned importance of making food composition data available to research, clinical practice, policymakers, and consumers, such a legal obligation is not expected in the near future. Until then, an approach that combines data provided by food manufacturers where available, and data collected in food monitoring studies, supplemented with data collected from other approaches, such as crowdsourcing and web scraping, is needed. Challenges in connecting different datasets need to be addressed with harmonization in data structure and the development of automatic services.

## Compilation of Branded Foods Database in Slovenia

### History of Monitoring Branded Foods in the Slovenian Food Supply

In Slovenia, data requirements about labeling and composition of branded foods were expressed in 2011, soon after the adoption of Regulation (EC) No 1924/2006 regarding nutrition and health claims made on food products (7). This regulation also provided for monitoring changes in the food supply, particularly the use of nutrition and health claims on foods. It should be mentioned that at that time, harmonized standards for such data collection were not yet available, but the importance of this research topic was highlighted in other countries. For example, Lalor et al. (40) published results of monitoring the Irish food supply in 2010. Their monitoring approach was used to set up food monitoring in Slovenia.

The first food monitoring study in Slovenia was conducted in 2011 within research project V7-1107 ‘Nutrition and health claims on foods’ (41), funded by the Slovenian Research Agency and the Ministry of Agriculture, Forestry, and Food of the Republic of Slovenia. Data collection was described previously (42). In short, researchers visited the grocery stores of three retailers (Mercator, Spar, Hofer) in Ljubljana, which covered the majority of the national food supply. In agreement with the retailers, data collection was done directly in grocery stores; researchers extracted food labeling information into an Structured Query Language (SQL) database, using local notepad computers. A special electronic form was developed, enabling the quick collection of the data into the database. European/International Article Number (EAN) barcodes were used as unique product identifiers. Such an approach avoided duplicates; each product was only collected once, even if it was available on different shelves or in different food stores. The selection of food categories was made according to Lalor et al. (40), with the addition of processed seafood, ready meals, vegetable oils, and plant-based milk/yogurt imitates. The dataset only contained numeric/text information, without any pictures. Altogether, 6,348 unique items were sampled in this study. The following information was collected: date/time stamp, store identification, product EAN/barcode number, food category, food name, manufacturer, use of health and other symbols, use of nutrition/health claims, and nutrition declaration information (energy, fat, saturates, sugar, sodium, fiber). At that time, the labeling of nutritional information on processed branded foods was not yet mandatory; calorie content was available for 65.6% of foods in the dataset, and sodium content for 39.2%.

Confidentiality agreements were signed with major retailers in exchange for their nationwide 12-month sales data. Sales data were provided by each food (EAN barcode number) separately. If the same product (barcode number) was sold at different retailers, the total sale volume was calculated. The barcode number was used to match food products in our dataset with corresponding sales data. Sales data were obtained for 80.4% of foods in our dataset (42).

A major challenge of the 2011 study was that the entire data collection process was conducted within food stores. Researchers had temporary collection points, with laptops connected with the SQL database over the Wi-Fi network. This approach presented a burden for retailers, who had specific requirements to limit the effect of the study on consumers. Another limitation of the monitoring approach was that the dataset produced did not enable further verification of the data or exploitation of parts of the food labeling which were not the subject of the original study. For example, post-data collection studies of marketing approaches to children were not able to be conducted because these were not included in the initial data collection.

Another monitoring approach was used in 2013 within the European FP7 research project CLYMBOL (“Role of health-related CLaims and sYMBOLs in consumer behavior”), funded by the European Commission (43, 44), which was also conducted in Slovenia. While the 2011 food monitoring study collected data for all available foods in the selected food categories, in the CLYMBOL project, foods were sampled with a randomization approach. Food was purchased in stores, and data collection was completed outside the food store (44). Because foods were perishable, all the packages were photographed to enable later processing. The strength of this approach was the ability to verify all recorded information, while limitations included logistic challenges in the randomization, purchasing, and data collection. Also, purchasing foods incurred unnecessary food waste and considerable costs, which were mitigated by donations. Altogether, 2,034 foods were sampled in five countries (about 400 per country) (44).

### Introduction of a Standardized Food Monitoring Approach

In 2015, the national “Nutrition and Public health” research program was initiated in Slovenia with funding from the Slovenian Research Agency. The 2011 branded food composition database had proved very useful for researchers and policymakers, so a major objective of this national research program was also to follow changes in the food supply, with a specific focus on processed branded foods (45), a key contributor in various diet-related non-communicable diseases (46). This time period was also marked by progress in the international harmonization of the methodological approach for conducting food monitoring studies (19, 23, 24), providing guidance for food categorization and data collection, and prioritizing data extraction from photographs of food labels.

A decision was therefore taken to develop an infrastructure to support more efficient food monitoring studies in Slovenia. At that time, the George Institute for Global Health (Australia) ‘FoodSwitch’ (47) and the University of Toronto (Canada) ‘Food Label Information Program (FLIP)’ (48) were examples of the most sophisticated infrastructure in this field, and their developers offered important insights, which supported the development of the infrastructure in Slovenia.

In line with this, the Composition and Labeling Information System (CLAS) infrastructure was developed, composed of:

- Data collection via the smartphone application CLAS for use by researchers in food stores; details are specified in Supplementary Table 1 and Supplementary Figure 1. This mobile application works on the Android operating system and directly communicates with the background CLAS SQL database over a Wi-Fi network or through mobile data transfer.
- Data extraction and management via the online CLAS tool; details are specified in Supplementary Table 2 and Supplementary Figure 2. The tool runs on MS Windows Server 2016, while the database runs on MS SQL Server 2016 Standard.

Both tools are interconnected, enabling easier data collection. The smartphone application CLAS can be used for real-time collection by several researchers at the same time without risk of duplication, as barcodes are used as a unique identifier for this purpose, thus ensuring data from a given food is only collected once. From inside food stores, the researcher scans the barcode of a product (using the CLAS application), and if data from this product has not yet been collected in the cross-sectional study, the mobile application requires the researcher to take photos of all sides of the food packaging and to input price information. Timestamp (date, time) and food store identification are saved automatically. All these data and images are directly sent from the mobile application to the online CLAS tool, where they are checked for quality using both automatic controls and a manual check of each product by the researcher (49). If a product with the same barcode was already collected in the same cross-sectional study, only the store identification and time-stamp are saved to the database, and the researcher is notified that product information is already collected, thus removing duplication of work. Data extraction is completed in an online CLAS tool, with the support of Optical Character Recognition (OCR) technology, and supported by manual work and cross-checking (49).

Since 2015, all food monitoring studies in Slovenia have been conducted with this approach, employing data extraction from food labeling photographs (Table 4). In 2015, food monitoring studies were conducted in the same three retailers as in 2011 (Mercator, Spar, Hofer), while retailers Tuš and Lidl were added in 2017 and Eurospin in 2020. Food monitoring in 2015 built on experiences gained in 2011 (42) and still included only selected food categories. A cross-sectional study in 2017 investigated almost all available pre-packed foods (excluding alcoholic beverages); however, alcoholic beverages were also included in 2020. A cross-sectional study in 2019 was conducted as a partial study, focusing only on food categories with higher sugar content, specifically addressing objectives of the national research project “Sugars in human nutrition: availability in foods, dietary intakes, and health effects” (51).

**Table 4:** Description of food monitoring studies conducted in Slovenia (2011–2020).

**Year**	**2011**	**2015**	**2017**	**2019**	**2020**
No. of records^*^	6,348	10,694	21,090	6,892	28,028
Included retailers	Mercator/Spar/ Hofer	Mercator/Spar/ Hofer	Mercator/Spar/ Tuš/Hofer/Lidl	Mercator/Spar/ Tuš/Hofer/Lidl	Mercator/Spar/ Tuš/Hofer/Lidl/ Eurospin
Sample	Selected food categories [details in (42)]	Selected food categories [details in (50)]	All food categories, excluding alcoholic drinks	Selected food categories [focus on categories with added sugar]	All food categories (including alcoholic drinks)
Nutrition declaration data	✓	✓	✓	✓	✓
Ingredient list data	*x*	✓	✓	✓	✓
Price	*x*	✓	✓	✓	✓
Notes	Manual collection of data from food labels in food stores.	Collection of data using CLAS infrastructure from pictures of food labels taken in food stores. All pictures are archived.			

* *Number of records before removing ineligible foods for specific studies/analyses (i.e., removal of items which combined toys, items with different types of foods in the same package)*.

As in the 2011 food monitoring study, arrangements were made with major retailers, who provided their nationwide 12-month sales data, and barcode numbers were used to match food products in the dataset with corresponding sales data.

**Figure 1:**
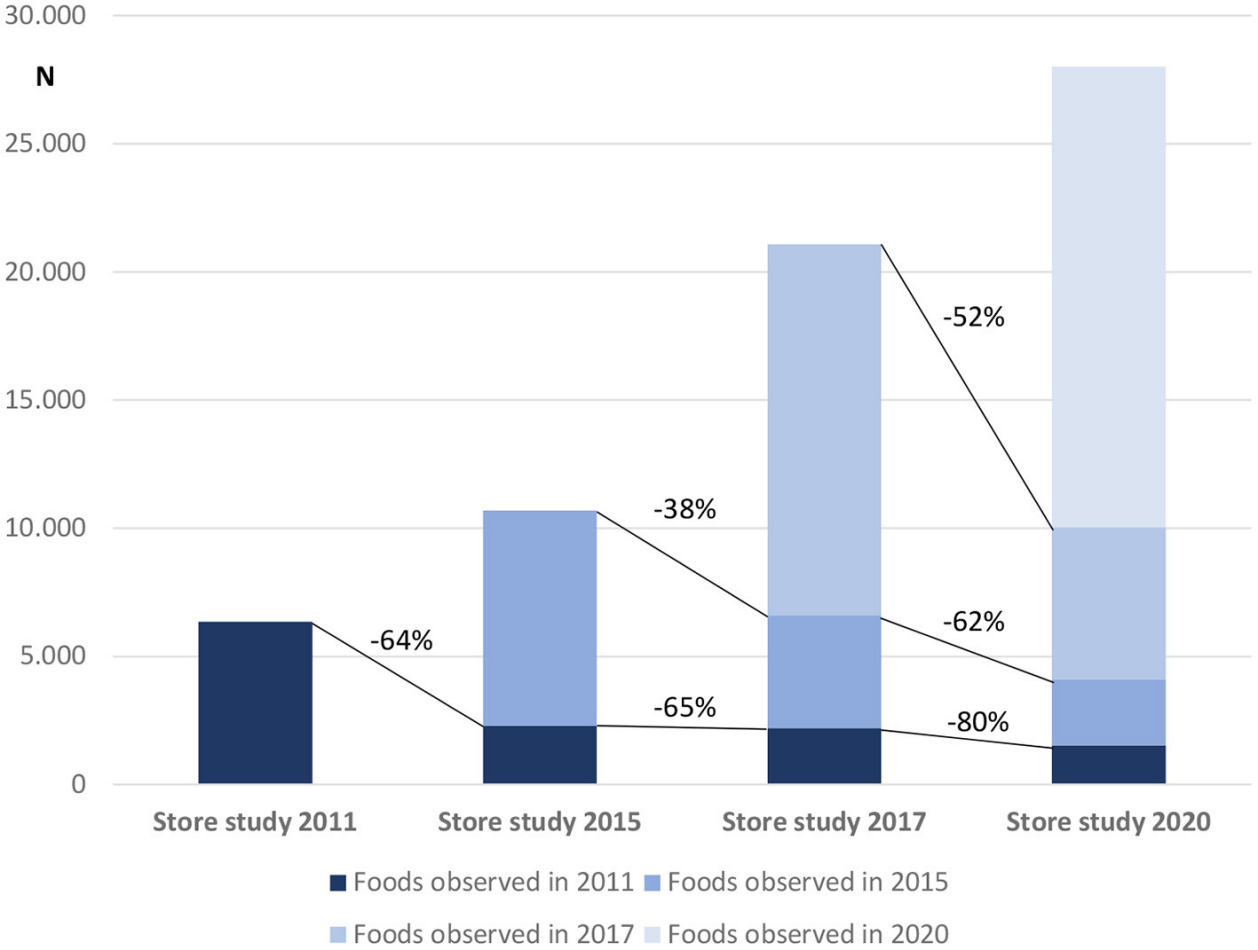
Number of foods collected in regular food monitoring studies in Slovenia.

In line with the increasing number of retailers in the food monitoring studies and the inclusion of additional food categories, the number of sampled foods has increased considerably since 2011; over 28,000 unique products were sampled in the last data collection (2020). With consideration that each regular data collection (2011, 2015, 2017, 2020) included the same/additional retailers/food categories, the compiled dataset can be used to provide interesting insights about the market-life of foods available in the food supply (Figure 1). For example, out of 6,348 products sampled in 2011, only 2,285 (−65%) were still found in 2015, and 1,526 (−80%) were found in 2020. Similarly, only about half of products from the complete food monitoring in 2017 were still on the market in 2020. These trends indicate that while a small proportion of branded foods in the food supply has a long market-life, many foods are quickly removed from the market and substituted with new products. Food supply studies are challenging because branded food databases become outdated very quickly. For this reason, monitoring is essential, and enforcing the obligation of sharing data openly for producers would be beneficial to the field.

### Introduction of Crowdsourcing

**Figure 2:**
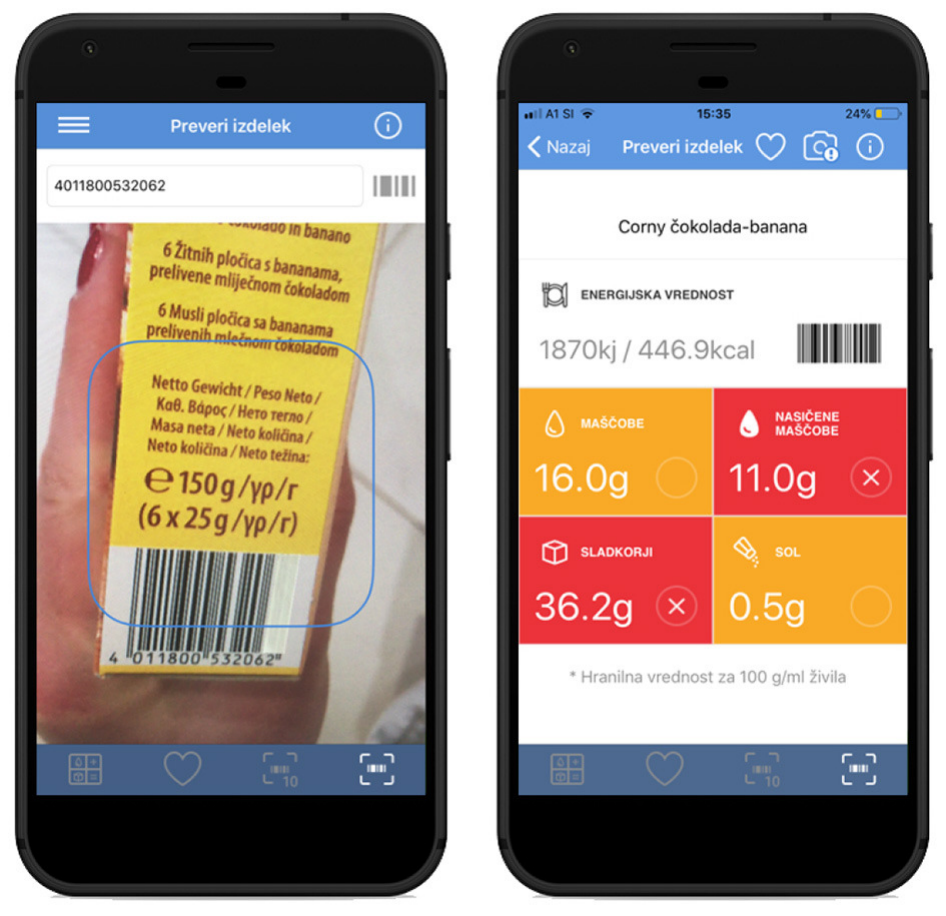
Branded food composition database can support consumers in interpreting food composition information. Example of the Slovenian smartphone application VešKajJeš (iOS/Android).

In 2019 a smartphone application called “VešKajJeš” (#VKJ; English translation: You know what you eat) was launched in Slovenia as part of the collaborative “IRIO” (‘Innovative solutions for informed choices') project. The application is available for Android and iOS platforms. The project was launched by the Nutrition Institute, ‘Jožef Stefan’ Institute, and the Slovenian Consumer Organization and supported by the Slovenian Ministry of Health. The app enables users (consumers) to scan the barcode (EAN) of selected food and receive feedback information on the product's nutritional composition. It also interprets the nutritional information based on the nutrient profile by using the food traffic light labeling system (Figure 2), supporting healthier food choices.

**Figure 3:**
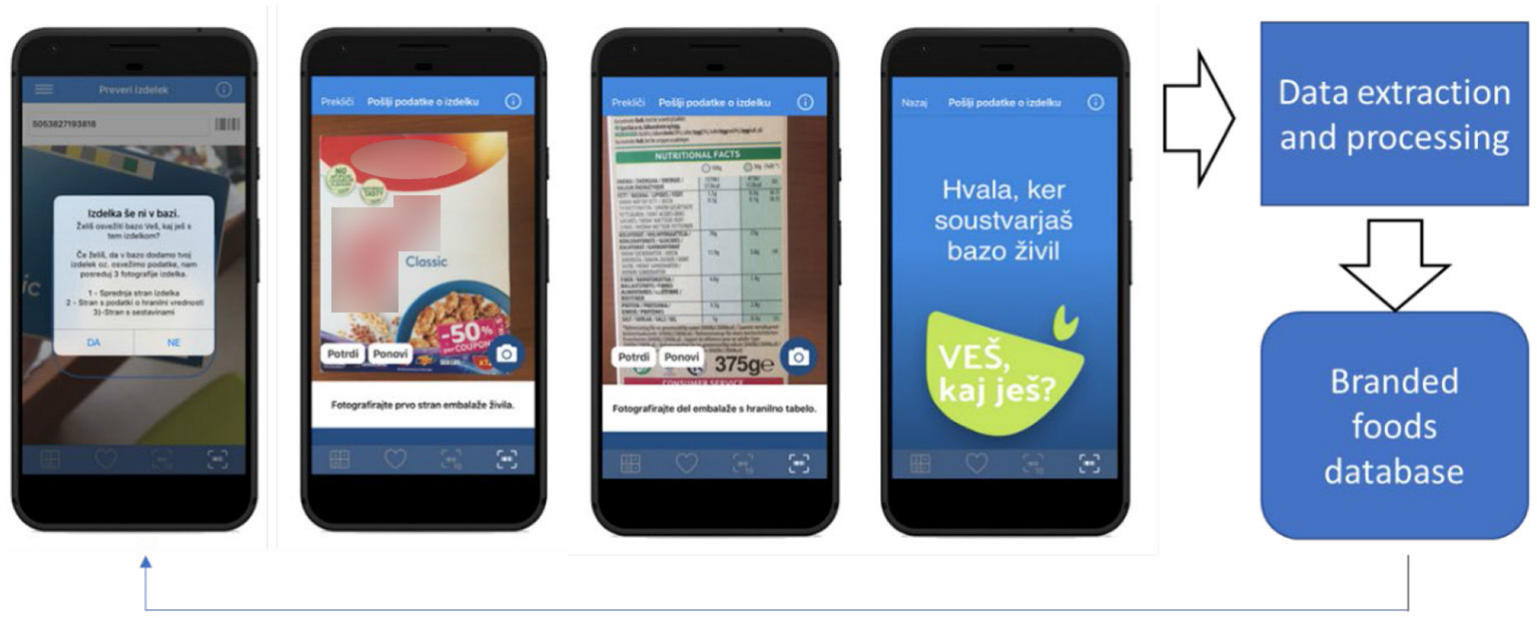
Crowdsourcing approach used in Slovenia with VešKajJeš smartphone application.

The crowdsourcing function of the application is activated when a user scans a food barcode that is either not yet included in the branded food database or when there is a difference between the nutritional composition of the scanned food and the information presented in the mobile application VešKajJeš (Figure 3).

Crowdsourcing information is stored and processed in a special web application (bazil.si), which was developed by the ‘Jožef Stefan’ Institute within the IRIO project (specified in Supplementary Table 3). Bazil.si enables researchers to view data sent by users and transcribe the data of interest from the labels that can be seen on the images. Products are processed based on search volume, so the products that are most scanned by users are added to the database as soon as possible to ensure a high rate of positive answers by the VešKajJeš application. Extracted parameters include EAN number, product name, category, and data from mandatory nutrition declarations. Since its release in mid-2019, the application users have contributed 11,482 unique items, 9,348 of which were processed. The application currently has 24,000 active users (over 1% of the whole population of Slovenia) who make approximately 250,000 inquiries per year. In general, the application is well-received by the general public, and was also highlighted on the Information Society Multiconference with ‘the information strawberry’ award for best IT achievement in year 2019 (52). It is worth mentioning that for the success of the application, a well-planned and executed media campaign was and still is crucial.

**Figure 4:**
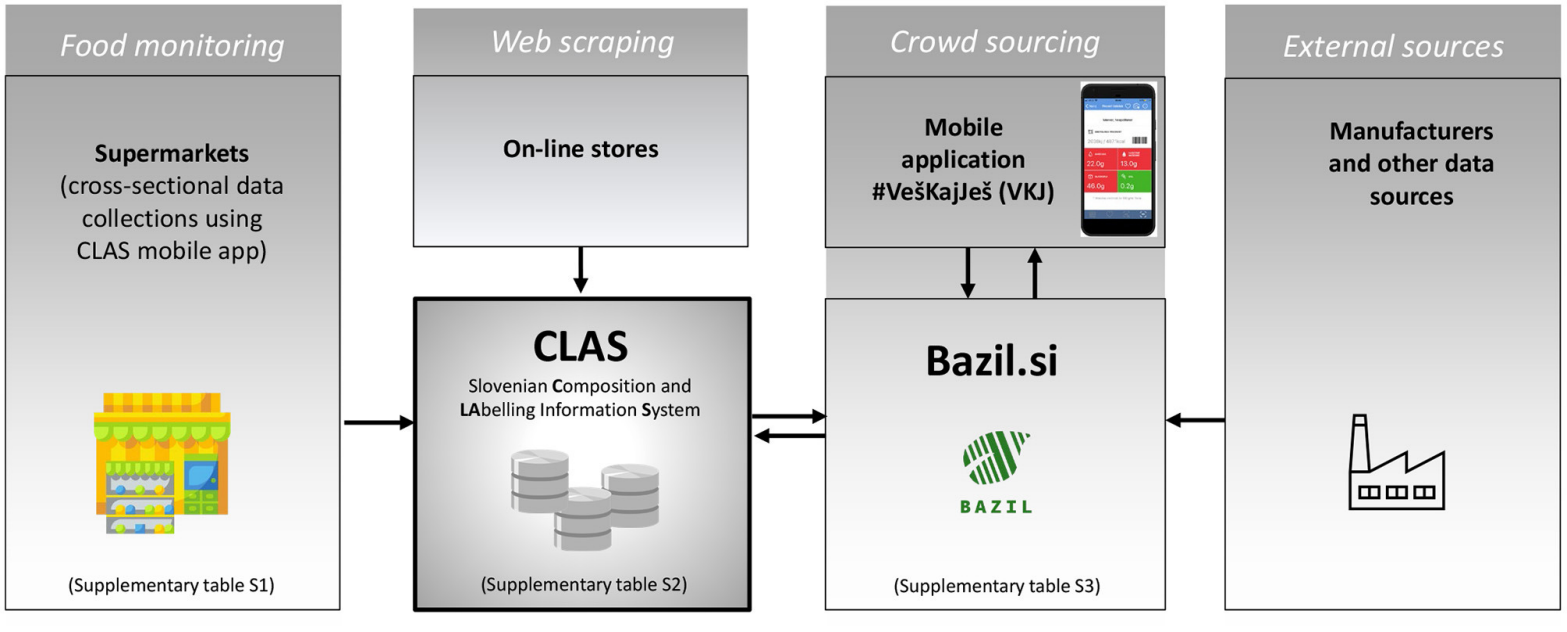
Schematic presentation of data-pathways in the Slovenian Composition and Labeling Information System (CLAS).

A dataset compiled from crowdsourcing is currently used as a complementary data resource for the mobile app #VKJ. This enables the composition of newly identified foods to be also available to other app users. This dataset is also used within the previously mentioned FNS Cloud project (39), which is investigating if crowdsourcing can be used as an efficient, effective, and low-cost method for generating reliable food composition datasets. Both CLAS and Bazil.si are inter-connected with Application Programming Interface (API) to support continuous data transfers (Figure 4).

## The Exploitation of Use of Branded Foods Database in Nutrition Research

The first Slovenian food monitoring study in 2011 identified a major challenge: many foods were not labeled with full nutrition declaration data. Considering that nutritional profiling of foods can be only be conducted if sufficient details on nutrition composition are available, a relevant question was whether such missing information could be supplemented from other sources. Eržen et al. (53) conducted a comparative evaluation of the use of generic food composition databases for nutrient profiling. The study was conducted with two nutrient profiling models (UK Ofcom, Australian FSANZ). Moderate/good agreement was observed for the dataset compiled from food labels, compared with the dataset compiled using the food matching technique (54). This was particularly important in the 2011 dataset, as, at that time, labeling of nutrition declaration was not yet mandatory in the European Union. However, the situation changed considerably after the implementation of harmonized EU food labeling Regulation (EU) No 1169/2011 (6) in December 2016, which made this information part of mandatory food labeling. Nevertheless, some types of information which are commonly used in nutrition research (i.e., content of dietary fiber) are still not part of mandatory food labeling, meaning that the food-matching approach is still relevant.

Datasets collected in Slovenian food monitoring studies were either directly or indirectly used in numerous studies in public health and nutrition research. We have reviewed all types of data exploitation, which resulted in peer-reviewed scientific publications. We identified *N* = 18 studies, focused on analyses of branded foods datasets (Table 5), and *N* = 11 studies in which branded foods datasets were used indirectly as a source for estimating the nutritional composition of branded foods collected in other studies (Supplementary Table 4).

**Table 5:** Examples of studies directly exploiting Slovenian branded food databases (2011-2020; details provided in Supplementary Table 4).

**Type of use**	**Examples**
Assessments of nutritional composition of food in the food supply	Salt/sodium (42, 49); Free/total sugar (50, 55)
Assessments of the use of specific ingredients	Partially hydrogenated vegetable oils/fats as sources of trans fatty acids (54); food fortification (56)
Assessments of the use of food additives	Sweeteners (51, 57), titanium dioxide (58)
Assessments using nutrient profiling approach	Methodological/assessment of the use of external data for missing values (53); international comparisons (2); nutrient profiling of the food supply (20, 59, 60)
Assessment of food marketing techniques on food labels	Nutrition and health claims on foods (41, 61), children marketing (62), gluten-free claim (63)

*List only include studies where Slovenian branded food databases were exploited directly. Food monitoring studies datasets were used for several other studies, for example, to support in the sampling of foods in the food supply for assessment of the content of trans fatty acids in foods (64, 65), for assessment of salt iodization (66), for assessment of dietary intakes (67–71), food marketing (25, 72–74) and offers in vending machines (75, 76). Studies exploiting additional Slovenian branded foods composition datasets (44, 66, 77–79) are also not included*.

### Studies Directly Exploiting Branded Food Databases

#### Assessments of Nutritional Composition of Food in the Food Supply

Several studies have focused on assessing the nutritional composition of processed foods available in the food supply using nutrition declaration information. Korošec et al. (42) used the 2011 dataset to investigate the importance of sales data in assessing the food supply. A similar study was also conducted on the 2015 dataset (49) to investigate time-changes in the sodium content in branded foods as a result of voluntary food reformulation activities. The study identified a trend of reduced sodium content in cheese and a neutral trend in bread and meat products, while higher sodium content was observed in ready meals, highlighting that additional efforts are needed for sodium reduction (49).

A similar approach was used by Zupanič et al. (50) on the 2015 dataset, but with a focus on total/free sugar content in branded foods. A particular challenge in this study was that in the EU, food labels only provide information on total sugar content, but not for added and/or free sugar. Therefore, an algorithm for estimating free sugar was established based on the protocol developed by Bernstein et al. (80), where labeled food ingredient information is used together with nutrition information data. Altogether, more than half of the products in the dataset contained free sugar; categories with the highest content were honey/syrups, jellies, chocolate and sweets, jam/spreads, and cereal bars. Using sales data, chocolate/sweets and soft drinks were identified as major contributors to free sugar in the food supply. In 2015, the Slovenian food industry accepted a pledge to lower sugar content in beverages. To investigate the efficacy of industry self-regulation on trends in free sugar content in branded foods, a similar study was conducted using the 2017 dataset (55). The study identified soft drinks as the most important source of free sugar, responsible for 28% of all free sugar sold with branded foods. Few positive trends were observed, and the study also highlighted that in the categories with the highest share of free sugar, brands with higher market share were commonly sweeter than category averages.

#### Assessments of the Use of Specific Ingredients, Including Additives

Food composition datasets were also explored with a specific focus on particular ingredients reported in labeled food ingredient lists. One particularly interesting food ingredient is partially hydrogenated oils (PHO), a source of trans fatty acids (TFAs), which is a key risk factor for cardiovascular disease (81). After a surprising report by Stender et al. (82), which identified that PHO-containing biscuits in Slovenia are still a source of TFAs, Zupanič et al. (54) investigated Slovenian branded foods datasets compiled in 2015 and 2017 to identify food categories where PHOs were most commonly used. In this study, biscuits were identified as a food category with the most frequent usage of PHO, but the study also showed a notably lower usage of this ingredient in 2017. Same approach has been used for the exploitation of food fortification practices with vitamin D (56).

Similarly, Hafner et al. investigated trends in the use of different artificial sweeteners in drinks in the Slovenian food supply in the years 2017, 2019 (51) and 2020 (57), while Blaznik et al. (58) exploited usage of titanium dioxide (food additive E 171) in the food supply. The latter case is an excellent example of the exploitation of branded food composition databases for rapid public health use, supporting food policers and food control authorities. Titanium dioxide has been used as a very efficient white food pigment for decades (83), but later research showed that a notable proportion of this additive are nanoparticles (84, 85) and highlighted genotoxicity concerns (86, 87). After a re-evaluation of the safety of this additive, the EFSA published an official opinion in May 2021 (88), concluding that because a concern for genotoxicity could not be excluded, this additive could not be considered safe anymore. As a rapid response, the last Slovenian branded foods dataset (CLAS 2020) was investigated for the use of TiO_2_ and compared with the 2017 dataset. Preprints of the study were published 1 month after the publication of the EFSA opinion in June 2021 (89), and a peer-reviewed publication was published in August 2021 (58). Chocolate and sweets, and chewing gums were identified as categories in which TiO_2_ is most commonly used; its use significantly decreased (by 50%) between 2017 and 2020.

#### Assessments Using Nutrient Profiling Approach

While previous examples investigated the food supply with a focus on specific nutrients or food ingredients, another approach employs nutrient profiling, in which several nutrients/constituents are considered at the same time. ‘Nutrient profiling’ is described as the science of classifying foods according to their nutritional composition for reasons related to preventing disease and promoting health (90, 91). We should note that there is no consensus on the superiority of a specific nutrient profiling system. Models are used for very different purposes (adjusting the food supply, food marketing, supporting healthy dietary choices, etc.) and also differ considerably in the profiling algorithms, i.e., in the number/selection of food categories, the involved nutrients and food constituents, and regarding involving of scoring (92–94). Two common general approaches are the use of category-based cut-off values, as in case the WHO Office for Europe nutrient profile (WHOE) (95), or a scoring approach with consideration of different nutrients/constituents, as in the case of the United Kingdom Food Standards Agency nutrient profiling system (NPS) (96), Australian Nutrient Profiling Scoring Criterion (NPSC) and Health Star Rating (HSR) (97), and French Nutriscore (NS) (98, 99).

Dunford et al. (2) used the 2015 version of the Slovenian branded foods dataset in an international comparison of the healthiness of packaged foods/beverages from 12 countries (Australia, Canada, Chile, China, India, Hong Kong, Mexico, New Zealand, Slovenia, South Africa, the UK, and the USA) using the HSR nutrient profiling system. Specific food categories were highlighted as particularly problematic in the Slovenian food supply, for example, beverages, snacks, and meat products. Altogether, Slovenia ranked in the middle between the countries with the highest overall nutrient profile (UK, USA, Australia, and Canada) and the countries with the lowest ranks (India, Hong Kong, China, and Chile) (2). The 2015 dataset was also used to compare different front-of-package labeling schemes (20) and investigate the nutritional quality of the foods labeled with health-related claims (59). The latter study reported that about 68% and 33% of the foods labeled with health-related claims passed NPSC and WHOE criteria, respectively, highlighting the need for stricter regulations to use such claims on foods.

**Figure 5:**
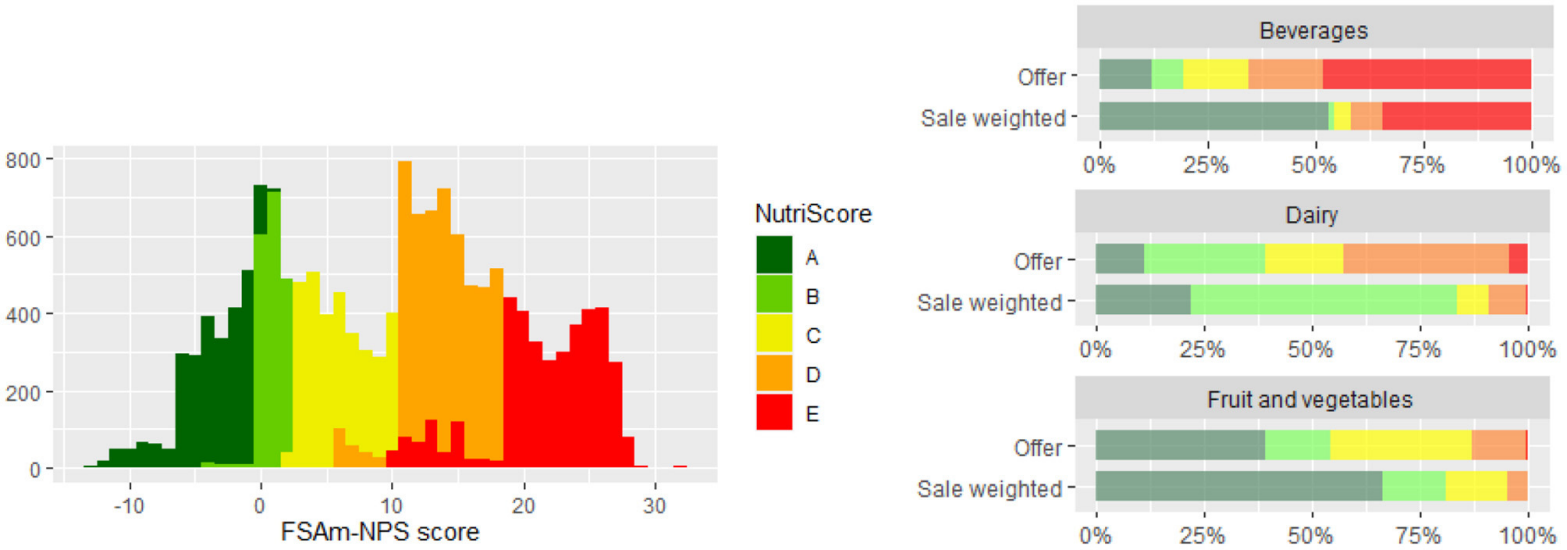
**(Left)**: Distribution of Food Standards Agency Nutrient Profile (FSAm-NPS) scores and corresponding Nutri-Score grades for foods and drinks in the Slovenian food supply. **(Right)**: Distribution of Nutri-Score grades across selected categories using sale-weighting. Reproduced from Hafner et al. (60) with approval of authors.

Nutrient profiles are a very timely topic because of the current discussions in the European Union on the possibility of implementing a mandatory front-of-pack nutritional labeling scheme. Nutri-Score (NS) is an example of such a scheme, which was originally developed in France (98, 99) but is currently voluntarily used in several other European countries (100). NS presents an upgrade of the NPS; it is a scoring system based on the content of selected nutrients/constituents per 100 g of food. This nutrient profile model grades the nutritional quality of products with a 5-color/letter scale from dark green (A; healthy) to dark orange (E; least healthy) (101). Very recently, Hafner et al. used the sale-weighting approach to evaluate the power of NS for discriminating the nutritional quality of branded foods using the 2017 branded foods dataset (60). The study indicated the very good ability of this nutrient profile model to compare between foods (Figure 5, left). This was confirmed also within food (sub)categories. Furthermore, it was shown that the availability of foods does not always reflect their sales; notable differences between available and sold foods were reported, particularly in beverages, dairy products, fruits, and vegetables (Figure 5, right).

#### Assessment of Food Marketing Techniques on Food Labels

In addition to the previously mentioned evaluation of the nutritional quality of food labeled with nutrition and health claims (59), several other studies also exploited branded food databases to assess food marketing techniques on food labels. The first such study employed the 2011 dataset to investigate consumers' exposure to nutrition/health claims on branded foods (41); 37 and 15% of foods were labeled with nutritional or health claims, respectively (this was 45 and 11%, after correction for market-shares). The majority of health claims were general non-specific and/or function claims. Later on, researchers exploited the 2015 dataset to investigate the use of gluten-free claims (63), heart images (61), and marketing to children (62). Studies identified several challenges of such poorly regulated marketing, which often promotes foods with questionable overall nutritional composition. For example, the use of cartoon characters and other children's marketing techniques on food labels was almost exclusively linked to unhealthy foods (62), indicating the need for regulation in this area.

### Indirect Use of Branded Food Databases in Nutrition Research

In addition to the above-mentioned direct exploitation of branded food databases, datasets have also been used indirectly to support nutrition research (Supplementary Table 3). A relevant example is supported in the sampling of foods in the food supply. Food labeling only includes some types of information, while others must be investigated using laboratory investigations. However, sampling in such studies is very challenging because the tens of thousands of foods that are available on the market cannot be easily analyzed. In such cases, branded foods datasets can be used to support the selection of foods for laboratory analyses. For example, Kušar et al. (64) and Mencin et al. (65) used the 2017 dataset to select branded foods for determining the number of trans fatty acids (TFA) analytically. Studies have identified specific food categories with higher content of TFAs, supporting the Slovenian government in implementing a regulatory ban on such foods (102).

We should also mention that branded foods datasets are a useful resource in conducting dietary surveys. In line with guidance on EU Menu Methodology (103), nationally representative food consumption studies should employ at least two 24-h recalls, meaning that the collected datasets contain thousands of different food items, for which nutritional composition needs to be estimated. While in some cases, this can be done using generic food databases, estimation of the composition of branded foods is much more reliable if branded food datasets are also employed. The Slovenian national dietary survey SI.Menu was conducted in 2017/2018 (104), and the 2017 edition of the branded foods dataset was used to estimate the nutritional composition of reported foods. This approach has already supported several epidemiological studies, which investigated diet-related-risk factors and population intakes of total/free sugars (69), dietary fiber (70), TFAs (68), vitamin D (67) and folate (71).

Studies investigating food marketing are another example of using branded food datasets for estimating the nutritional composition of processed foods. Such studies typically result in very large collections of advertisements (i.e., from television, magazines), which are linked to specific foods. Considering that majority of food marketing is for branded foods (2), such products can be linked with branded food databases while missing information is searched in other sources. Such an approach was used in analyses of food marketing to children in magazines (73) and on television (25, 72). Considering that television marketing was identified as particularly problematic, the Slovenian government introduced regulatory restrictions for marketing within programs targeting children, and this intervention was also evaluated (74) using a branded food database.

Similarly, the nutritional quality of foods available in vending machines was also investigated. Rozman et al. reported the poor nutritional quality of snacks (75) and beverages (76) available in vending machines in health and social care institutions in Slovenia.

## Challenges and Conclusions

Branded foods databases are becoming valuable not only in nutrition research but also for clinical practice, policymakers, businesses, and end-users. In contrast to generic foods, branded foods are marked by rapid changes in the food supply because of reformulations, the introduction of new foods, and the removal of existing ones from the market. Also, different branded foods are available in different countries. This not only complicates the compilation of branded foods datasets but also makes such datasets out-of-date quite quickly.

In an ideal situation, the composition of branded foods would be provided in an open-access format automatically by food manufacturers, like in the Netherlands. There is no indication that such data sharing will become mandatory in the near future; therefore, branded food databases also need to employ other approaches to assure that data are up-to-date. A standard approach is conducting food monitoring studies in supermarkets to collect the information presented on food labels. Such an approach has several challenges: (1) studies can be only conducted periodically in selected stores, (2) sophisticated infrastructure is needed to conduct such studies efficiently, (3) depending on the extent of the data collected, such studies are related to notable man-power and costs, (4) a long time period is needed to complete such studies: when data-collection and analyses are completed, the situation in the food supply could already be different, (5) the accuracy of the data is limited to the accuracy of the data provided on food labels. Therefore, complementary approaches are used to supplement such cross-sectional datasets, for example, more regular data collections of the (much more limited) offer of branded foods in online stores and crowdsourcing to collect data about the most relevant foods in real-time.

Branded food databases can be used directly for assessments of nutritional composition of food in the food supply (i.e., salt, sugar content), use of specific ingredients, for example, food additives, for nutrient profiling, and the assessment of marketing techniques on food labels. Such datasets are also valuable for other studies, for example, for nutrient intake assessments in dietary surveys.

## Author Contributions

IP: conceptualization and first draft. IP, MH, KŽ, BB, BK, and AK: manuscript writing—review and editing. All authors have read and agreed to the published version of the manuscript.

## Funding

In Slovenia Composition and Labeling Information System (CLAS) has been conducted by the Nutrition Institute (Ljubljana, Slovenia) within the national research programme “Nutrition and Public Health” (P3-0395) and “Infrastructure programme for monitoring of the composition and labelling of foods” (IO-0054), funded by the Slovenian Research Agency. Development of the infrastructure and data collections were further supported by several national research projects (L7-1849, V3-1901, L4-9305, L3-9290, L4-7552, L3-7538, V3-1501, V7-1107, P2-0098), funded by Slovenian Research Agency, the Ministry of Health of Republic of Slovenia, and Ministry of Agriculture, Forestry, and Food of the Republic of Slovenia; by national health promotion programmes (IRIO/mobile application VešKajJeš/Do you know what you eat? and Do you know what you drink?), funded by the Ministry of Health of Republic of Slovenia, and by the Food Nutrition Security Cloud project (FNS-Cloud), which received funding from the European Union's Horizon 2020 Research and Innovation programme (H2020-EU.3.2.2.3.—A sustainable and competitive agri-food industry) under grant agreement no. 863059. Information and views in this report do not necessarily reflect the official opinion or position of the European Union. Neither European Union institutions and bodies, nor any person acting on their behalf, may be held responsible for the use that may be made of the information contained herein.

## Conflict of Interest

IP has led and participated in various other research projects in the area of nutrition, public health, and food technology, which were (co) funded by the Slovenian Research Agency, Ministry of Health of the Republic of Slovenia, the Ministry of Agriculture, Forestry, and Food of the Republic of Slovenia, and in the case of specific applied research projects, also by food businesses. The remaining authors declare that the research was conducted in the absence of any commercial or financial relationships that could be construed as a potential conflict of interest.

## Publisher's Note

All claims expressed in this article are solely those of the authors and do not necessarily represent those of their affiliated organizations, or those of the publisher, the editors and the reviewers. Any product that may be evaluated in this article, or claim that may be made by its manufacturer, is not guaranteed or endorsed by the publisher.
