# Supplementary material for: Branded Foods Databases as a Tool to Support Nutrition Research and Monitoring of the Food Supply: Insights From the Slovenian Composition and Labeling Information System
Source: Front Nutr. 2022 Jan 4;8:798576. doi: 10.3389/fnut.2021.798576 (PMC8763694; doi:10.3389/fnut.2021.798576)
Supplement: Supplementary file 1 [file Data_Sheet_1.docx]

**Supplementary Table S1:** Presentation of information collected with mobile application CLAS in food stores

| **Chapter** | **Information description** | **Notes** |
| --- | --- | --- |
| Registration | User ID | Each user (researcher) logs in with unique registration identification (e-mail, password). |
| Store selection | Food store | Before collection, user select food store where food monitoring is taking place. |
| Scanning tool | Barcode | Barcode of prepacked food is taken with smart phone camera (automatic recognition of barcode number). If product with same barcode is already in the cross-sectional study database, researcher is informed about that (no pictures needed). New product can be still added manually. |
| Data collection | Product information | Time-stamp (date, time) and store ID is logged to the CLAS database automatically. Researcher is required to insert product price in EUR (VAT included). Product information are transferred to CLAS server in real time. |
| Picture collection | Labeling pictures | Product is photographed from all relevant sides (containing labeling information), starting with front-of-package picture. Pictures are transferred to CLAS server after confirmation. |

**Supplementary Table S2:** Presentation of information collected in the 2017 edition of the Slovenian Composition and Labelling Information System (CLAS)

| **Chapter** | **Information description** | **Notes** |
| --- | --- | --- |
| Description | Photographs (JPG) of all sides of food labeling | Among all available images specific pictures are identified: (1) front of package image; (2) nutrition declaration; (3) ingredients |
|  | Brand and Product name |  |
|  | Producer |  |
|  | Packaging quantity | Kg; L |
|  | Food category | GFMG food categories (19) are used for categorization |
|  | Issue | Notes on any potential quality issues, that need to be addressed |
| Nutrition | Information is food is ready to eat, or if preparation is needed. | For foods that need preparation, it is also indicated if labeled nutrition declaration is provided for product (1) as sold, or (2) for prepared food. |
|  | Information to which weight/volume nutrition declaration is provided | Typically, this is 100 g or mL (preferred choice), in some cases nutrition declaration is provided for serving size only. |
|  | Information from nutrition declaration | energy (kJ); fat (g); saturates (g); carbohydrate (g); sugars (g); fibre (g); protein (g); salt (g); mono-unsaturates (g); polyunsaturates (g); polyols (g); starch (g), vitamins and minerals as provided in Reg (EU) No 1169/2011 (6). |
| Ingredients | Ingredient list (text) | Additionally, presence of specific ingredients is followed in structured (yes/now) to enable quick data analyses (added sugar, dextrose/glucose/grape sugar; glucose syrup; glucose-fructose syrup, inverted sugar syrup, fructose/fructose syrup; lactose/maltose/other mono-, di- saccharides; maltodextrins; other sugar substitutes (honey, agave syrup, ...); fully hydrogenated oils/fats; (partly) hydrogenated oils/fats |
| Symbols | Information about presence of different symbols on food labels | We have preselected specific symbols followed on food labels, assigning production type, quality and nutrition/health references (examples provided below). If food label contains a health symbol not provided on our list, this is also indicated.  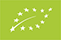  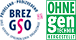  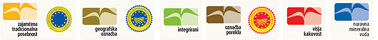  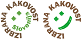  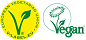  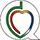 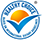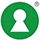  Other health symbol. |
| Claims | Presence of specific claims is indicated (yes/no) | - Nutrition claims - Health claims - Children marketing - Lactose free - Gluten free / Very low gluten - Contains caffeine (amount on caffeine content in mg per 100 g/mL) - Contains sweeteners - Contains aspartame (E951) - Contains liquorice |
| Nutrition claims | Information of all used nutrition claims | Nutrition claim wording/description, presentation (text/picture); position (front/back of package); regulatory classification according to Reg. 1924/2006 (7); referring component (energy or specific food constituent assigned); content of the referring component in food; regulatory claim status. |
| Health claims | Information of all used nutrition claims | Health claim wording/description, referring component (whole food or specific food constituent assigned); health relationship (WHO classification); regulatory classification according to Reg. 1924/2006 (7); presentation (text/picture); position (front/back of package); content of the referring component in food; regulatory claim status. |
| Allergens | Information on presence (contain/may contain/does not contain) of substances or products causing allergies or intolerances | Information provided for all substances/foods mentioned in the Annex II of the Reg (EU) No 1169/2011 (6):   - wheat / rye / barley / oats / unspecified cereals containing gluten - crustaceans - eggs - fish - peanuts - soybeans - milk - almonds / hazelnuts / walnuts / cashews /pecan nuts / brazil nuts / pistachio nuts / macadamia / unspecified nuts - celery - mustard - sesame seeds - sulfur dioxide/sulfite - lupin - molluscs |
| Additives | Information on all used food additives | Additive (E number and name; from the register of authorized food additives), additive function (categories as in PART C of the Reg (EU) No 1169/2011 (6)), regulatory status. |
| Vitamins & minerals | Information on all added vitamins and minerals | Constituent (specific vitamin/mineral); Source/chemical form (natural source or specific source/chemical ingredient from the European Community Register on the addition of vitamins and minerals and of certain other substances to foods (107); regulatory status. |

**Supplementary Table S3:** Presentation of information in web application Bazil.si

| **Chapter** | **Information description** | **Notes** |
| --- | --- | --- |
| Product | Photographs (JPG) of front of package, ingredients, and nutrition declaration |  |
|  | Status/ data on product addition to the database | Who added the product, time and date off product scanning, number of scanning by consumers |
|  | Global Trade Item Number |  |
|  | Brand and Product name |  |
|  | Food category |  |
|  | Packaging quantity (g) |  |
|  | Portion |  |
|  | Producer |  |
|  | Distributer |  |
|  | Country of origin |  |
|  | VešKajJeš status | 0: no profiling; 1: profiling |
| Ingredients | Ingredient list (text) |  |
| Nutrition declaration | Is the product labeled with nutrition declaration | Yes/ no, notes |
|  | Information to which weight/volume nutrition declaration is provided | Typically, this is 100 g or mL (preferred choice), in some cases nutrition declaration is provided for serving size only. |
|  | Information from nutrition declaration on macro in micronutrients | energy (kJ); fat (g); saturates (g); carbohydrate (g); sugars (g); fibre (g); protein (g); salt (g); mono-unsaturates (g); polyunsaturates (g); polyols (g); starch (g), vitamins and minerals as provided in Reg (EU) No 1169/2011 (6). |
| Additional ingredients | Content of additional ingredients of interest | Alcohol degree (%), caffeine content (mg), is the product containing (mark if yes) caffein, sweeteners or alcohol |
| Allergens | Information on presence (contain/may contain/does not contain) of substances or products causing allergies or intolerances | Information provided for all substances/foods mentioned in the Annex II of the Reg (EU) No 1169/2011 (6):   - wheat / rye / barley / oats / unspecified cereals containing gluten - crustaceans - eggs - fish - peanuts - soybeans - milk - almonds / hazelnuts / walnuts / cashews /pecan nuts / Brazil nuts / pistachio nuts / macadamia / unspecified nuts - celery - mustard - sesame seeds - sulfur dioxide/sulfite - lupin - molluscs |

**Supplementary Table S4:** Summary of studies using datasets compiled in Slovenian food monitoring studies (2011-2020)

| **Reference** | **Description** | **2011** | **2015** | **2017** | **2019** | **2020** |
| --- | --- | --- | --- | --- | --- | --- |
| **Assessments of nutritional composition of food in the food supply (nutrition declaration information)** | | | | | | |
| Korosec, 2014 (42) | Assessing the average sodium content of prepacked foods with nutrition declarations: the importance of sales data | 🗹 |  |  |  |  |
| Pravst, 2017 (49) | Changes in average sodium content of prepacked foods in Slovenia during 2011-2015 | 🗹 | 🗹 |  |  |  |
| Zupanič, 2018 (50) | Total and free sugar content of pre-packaged foods and non-alcoholic beverages in Slovenia |  | 🗹 |  |  |  |
| Zupanic, 2019 (55) | Free sugar content in pre-packaged products: does voluntary product reformulation work in practice |  | 🗹 | 🗹 |  |  |
| **Assessments of the use of specific ingredients (ingredients list information)** | | | | | | |
| Zupanič, 2018 (54) | Limiting trans fats in foods: use of partially hydrogenated vegetable oils in prepacked foods in Slovenia |  | 🗹 | 🗹 |  |  |
| Krušič, 2021 (56) | Use of branded food composition databases for the exploitation of food fortification practices: A case study on vitamin D in the Slovenian food supply |  |  | 🗹 |  | 🗹 |
| **Assessments of the use of food additives (ingredients list information)** | | | | | | |
| Hafner, 2021 (51) | Trends in the use of low and no-calorie sweeteners in non-alcoholic beverages in Slovenia |  |  | 🗹 | 🗹 |  |
| Hafner, 2021 (57) | The Sharp Rise in the Use of Low- and No-calorie Sweeteners in Non-Alcoholic Beverages in Slovenia: An Update Based on 2020 data |  |  | 🗹 | 🗹 | 🗹 |
| Blaznik, 2021 (58) | Use of food additive titanium dioxide (E171) before the introduction of regulatory restrictions due to concern for genotoxicity |  |  | 🗹 |  | 🗹 |
| **Assessments using nutrient profiling approach** | | | | | | |
| Eržen, 2015 (53) | A comparative evaluation of the use of a food composition database and nutrition declarations for nutrient profiling | 🗹 |  |  |  |  |
| Dunford, 2019 (2) | A comparison of the healthiness of packaged foods and beverages from 12 countries using the Health Star Rating nutrient profiling system |  | 🗹 |  |  |  |
| Pivk Kupirovič, 2019 (59) | Nutrient profiling is needed to improve the nutritional quality of the foods labelled with health-related claims |  | 🗹 |  |  |  |
| Pivk Kupirovič, 2020 (20) | Facilitating consumers choice of healthier foods: a comparison of different front-of-package labelling schemes using Slovenian food supply database |  | 🗹 |  |  |  |
| Hafner, 2021 (60) | Evaluation of the ability of Nutri-Score to discriminate the nutritional quality of prepacked foods using a sale-weighting approach |  |  | 🗹 |  |  |
| **Assessment of food marketing techniques on food labels** | | | | | | |
| Pravst, 2015 (41) | Consumers’ exposure to nutrition and health claims on pre-packed foods: Use of sales weighting for assessing the food supply in Slovenia | 🗹 |  |  |  |  |
| Lavriša, 2019 (62) | Marketing of foods to children through food packaging is almost exclusively linked to unhealthy foods |  | 🗹 |  |  |  |
| Lavriša, 2020 (63) | Nutritional composition of gluten-free labelled foods in the Slovenian food supply |  | 🗹 |  |  |  |
| Miklavec, 2021 (61) | Heart images on food labels: a health claim or not? |  | 🗹 |  |  |  |
| **Indirect usage of branded food datasets: support in sampling of foods in the food supply** | | | | | | |
| Kusar, 2021 (64) | Assessment of trans-fatty acid content in a sample of foods from the Slovenian food supply using a sales-weighting approach |  |  | 🗹 |  |  |
| Mencin, 2021 (65) | Content of trans-fatty acid isomers in bakery products on the Slovenian market |  |  | 🗹 |  |  |
| **Indirect usage of branded food datasets: Analyses of secondary samples – dietary surveys** | | | | | | |
| Zupanič, 2020 (69) | Total and free sugars consumption in a Slovenian population representative sample |  |  | 🗹 |  |  |
| Zupanič, 2021 (68) | Dietary intake of trans fatty acids in the Slovenian population |  |  | 🗹 |  |  |
| Hribar, 2021 (67) | Vitamin D intake in Slovenian adolescents, adults, and the elderly population |  |  | 🗹 |  |  |
| Koroušić Seljak, 2021(70) | Inadequate intake of dietary fibre in adolescents, adults, and elderlies: Results of Slovenian representative SI.Menu study |  |  | 🗹 |  |  |
| Pravst, 2021(71) | Dietary intake of folate and assessment of the folate deficiency prevalence in Slovenia using serum biomarkers |  |  | 🗹 |  |  |
| **Indirect usage of branded food datasets: Analyses of secondary samples – food marketing** | | | | | | |
| Korosec, 2016 (74) | Television food advertising to children in Slovenia: analyses using a large 12-month advertising dataset |  | 🗹 |  |  |  |
| Lavrisa, 2018 (72) | Trends in marketing foods to children in Slovenian magazines: a content analysis | 🗹 |  | 🗹 |  |  |
| Kelly, 2019 (25) | Global benchmarking of children's exposure to television advertising of unhealthy foods and beverages across 22 countries |  | 🗹 |  |  |  |
| Lavrisa, 2020 (74) | Regulating children's exposure to food marketing on television: are the restrictions during children's programmes enough? |  |  | 🗹 |  |  |
| **Indirect usage of branded food datasets: Analyses of secondary samples – vending machines** | | | | | | |
| Rozman, 2020 (75) | Sweet, fat and salty: snacks in vending machines in health and social care institutions in Slovenia |  |  | 🗹 |  |  |
| Rozman, 2021 (76) | Nutritional quality of beverages available in vending machines in health and social care institutions: do we really want such offers |  |  | 🗹 |  |  |

**Notes**: Studies exploiting additional Slovenian branded foods composition datasets (44, 66, 77-79) are also not included.


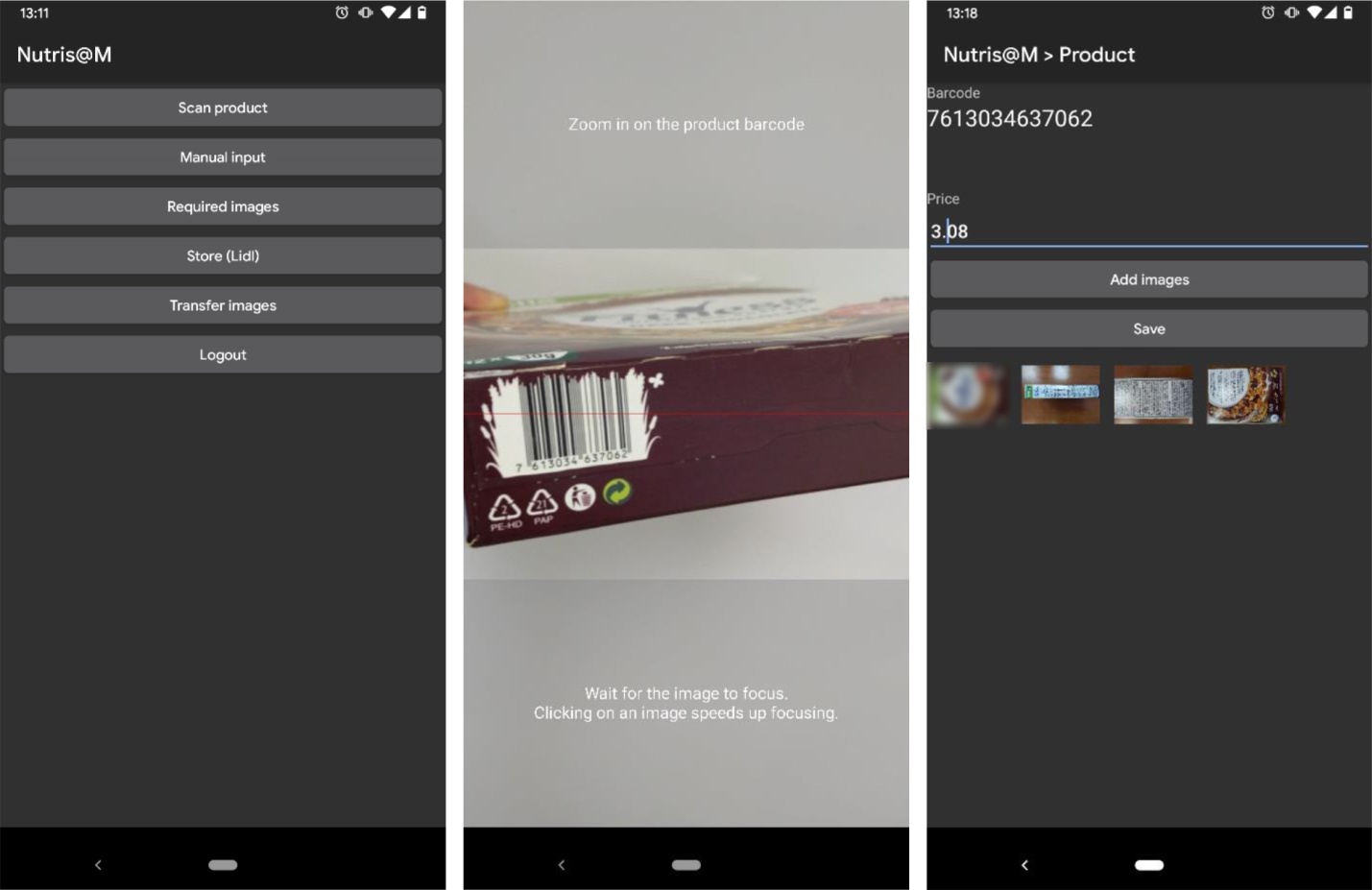


**Supplementary Figure S1:** Print-screens of the mobile application CLAS for data collection in food stores


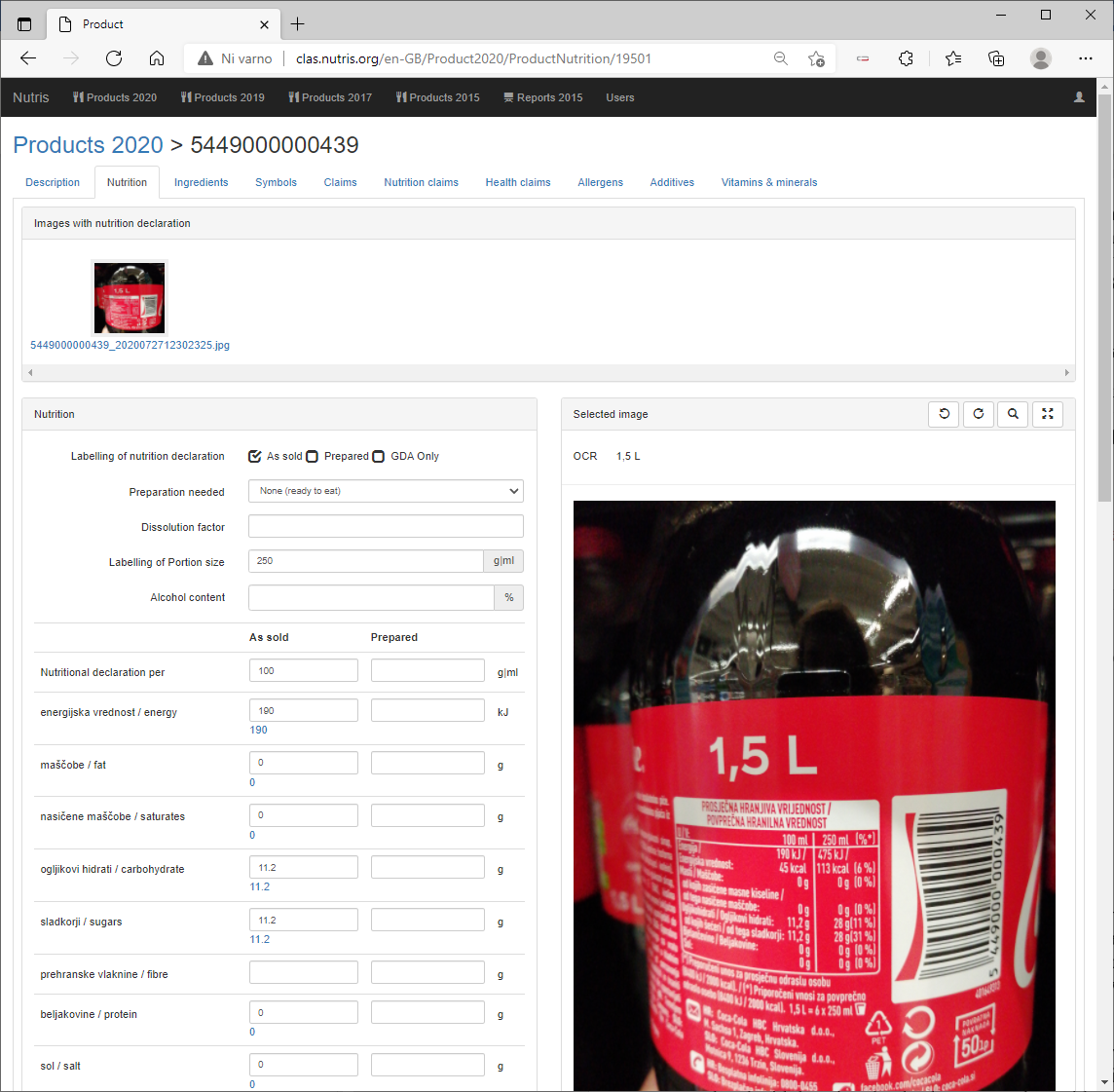


**Supplementary Figure S2:** Print-screen of the chapter of the on-line CLAS tool in the section of nutrition declaration information
